# Cellular and Biochemical Mechanisms of the Retroviral Restriction Factor SAMHD1

**DOI:** 10.1155/2013/728392

**Published:** 2013-07-17

**Authors:** Li Wu

**Affiliations:** Center for Retrovirus Research, Department of Veterinary Biosciences and Department of Microbial Infection and Immunity, The Ohio State University, 1900 Coffey Road, Columbus, OH 43210, USA

## Abstract

Replication of HIV-1 and other retroviruses is dependent on numerous host proteins in the cells. Some of the host proteins, however, function as restriction factors to block retroviral infection of target cells. The host protein SAMHD1 has been identified as the first mammalian deoxynucleoside triphosphate triphosphohydrolase (dNTPase), which blocks the infection of HIV-1 and other retroviruses in non-cycling immune cells. SAMHD1 protein is highly expressed in human myeloid-lineage cells and CD4^+^ T-lymphocytes, but its retroviral restriction function is only observed in noncycling cells. Recent studies have revealed biochemical mechanisms of SAMHD1-mediated retroviral restriction. In this review, the latest progress on SAMHD1 research is summarized and the mechanisms by which SAMHD1 mediates retroviral restriction are analyzed. Although the physiological function of SAMHD1 is largely unknown, this review provides perspectives about the role of endogenous SAMHD1 protein in maintaining normal cellular function, such as nucleic acid metabolism and the proliferation of cells.

## 1. Introduction

HIV-1 infection of human target cells is highly dependent on numerous host proteins to support viral replication [1, 2]. Genome-wide functional screens of HIV-1 cofactors have revealed that over two hundreds of human proteins are required for efficient HIV-1 replication in the cells [3–6]. However, during the long history of host-retrovirus interactions, primates including humans have evolved intrinsic immunity to defend against viral infection through host proteins called restriction factors, which often can be counteracted by viral proteins via intracellular degradation [7–9]. Studying those host restriction factors not only helps define the unique function of certain viral proteins in the infection and viral pathogenesis but also provides new insights into developing more effective intervention strategies to block retroviral infections.

The major HIV-1 target cells supporting viral replication are activated CD4^+^ T-lymphocytes [10], while other cell types such as primary monocytes, dendritic cells (DCs), and macrophages also contribute to initial infection and viral transmission [11, 12]. Nondividing CD4^+^ T-lymphocytes and myeloid cells including monocytes, DCs, and macrophages play an important role in establishment of HIV-1 initial infection and viral transmission [10, 11]. However, these cells are refractory to postentry HIV-1 infection due to multifaceted cellular mechanisms [11, 12]. Resting CD4^+^ T-cells are essential for the establishment and maintenance of HIV-1 latency as they can generate viral reservoirs in AIDS patients treated with antiretroviral drugs [13]. As a group of important antigen presenting cells to bridge the innate and adaptive immune responses, DCs can sense HIV-1 infection and trigger innate immunity against HIV-1 [14]. It is conceivable that restriction to HIV-1 infection of myeloid cells and resting CD4^+^ T-lymphocytes is a means of innate or intrinsic immunity against retroviral infection *in vivo* [15]. Recent studies have provided new insights into the molecular mechanisms underlying the viral defense in these important immune cells.

Several previous studies have identified and characterized three major retrovirus restriction factors, including APOBEC3G [16], TRIM5*α* [17], and tetherin (as known as BST2, CD317, or HM1.24) [18, 19]. Interestingly, HIV-1 has evolved effective ways to evade host restriction factors through its several accessory proteins, such as Vif to counteract APOBEC3G and Vpu to degrade tetherin [8, 20]. Recent studies have identified a novel retroviral restriction factor named SAMHD1 in non-cycling myeloid cells and resting CD4^+^ T-lymphocytes [21–25]. Human SAMHD1 has been shown to have a broad restriction of diverse retroviruses [26, 27]. In addition to HIV-1, SAMHD1 also blocks infection of other retroviruses, including HIV-2, feline immunodeficiency virus, bovine immunodeficiency virus, equine infectious anemia virus, and murine leukemia viruses [26, 27]. However, the mechanisms of SAMHD1-mediated retrovirus restriction and its physiological functions remain to be elucidated. Previously published reviews or commentaries have summarized the role of SAMHD1 in restricting HIV-1 infection in nondividing human immune cells [15, 28–34]. In this review, I would like to focus on the analysis of the cellular and molecular mechanisms by which SAMHD1 mediates retroviral restriction. I will also discuss potential cellular function of SAMHD1 in nucleic acid metabolism and the proliferation of cells.

## 2. The Identification and Characterization of SAMHD1 as an HIV-1 Restriction Factor

### 2.1. The Vpx Proteins from SIV and HIV-2 Enhance HIV-1 Infection in Myeloid Cells

Goujon et al. showed that preincubation with virus-like particles derived from sooty mangabey lineage SIV (SIVsm) significantly enhances transduction efficiency of human monocyte-derived DCs using HIV-1-derived lentiviral vectors [35]. Similarly, Vpx proteins from SIVsm and HIV-2 significantly promote HIV-1 infection in human monocytes and derived DCs and macrophages by facilitating the accumulation of full-length HIV-1 cDNA [36–40]. It has also been known that Vpx is critical for HIV-2 infection in human macrophages by enhancing the efficiency of viral reverse transcription [41]. Further studies indicated that Vpx from SIV or HIV-2 can interact with the host protein DCAF1 in the CUL4A/DDB1 and E3 ubiquitin ligase complex [42, 43], indicating that Vpx may target a putative HIV-1 restriction factor for proteasomal degradation in myeloid cells through the E3 ubiquitin ligase complex (for reviews, please refer to [44, 45]). These studies on Vpx and HIV-1 infection of myeloid cells have prompted the identification of SAMHD1 as an HIV-1 restriction factor in human myeloid cells [21–23].

Although HIV-1 genome does not encode the Vpx protein, Vpx has been a useful tool in the design of lentiviral vectors to better transduce myeloid-lineage cell types that are relatively resistant to lentiviral infection or transduction [46]. Sunseri et al. generated a modified HIV-1 to package SIV Vpx, which efficiently infects human primary macrophages and DCs [47]. Because Vpx is packaged into virions through the specific interaction with the p6 carboxy-terminal domain of Gag, the authors introduced the Vpx packaging motif of SIV p6 into an HIV-1 proviral DNA construct. The chimeric HIV-1 packaged Vpx *in trans* and showed significantly higher infectivity in DCs and macrophages compared with the wild-type virus. Furthermore, by introducing the Vpx coding sequence into the proviral DNA to incorporate Vpx *in cis, *the modified HIV-1 can efficiently replicate in DCs and macrophages [47]. The Vpx-containing HIV-1 could be more efficiently transmitted from DCs to cocultured CD4^+^ T cells, suggesting that Vpx may facilitate DC-mediated transmission of HIV-2 or SIV *in vivo*. Given that the infection of DCs with the Vpx-containing HIV-1 triggers activation of DCs and cocultured CD4^+^ T-cells [47, 48], these studies imply that the chimeric HIV-1 might be used to design DC-based vaccines to induce an enhanced innate immune response.

### 2.2. The Discovery of SAMHD1 as an HIV-1 Restriction Factor Interacting with Vpx

The identification of SAMHD1 was mainly dependent on the isolation and analysis of Vpx-interacting host proteins by mass spectrometry [21–23]. Previous studies have demonstrated that phorbol myristic acid- (PMA-) differentiated THP-1 cells can be rendered more permissive to HIV-1 infection by transduction of SIVsm/HIV-2 Vpx-containing virus-like particles derived from SIVmac [44]. Based on these observations, Laguette et al. identified SAMHD1 as a novel Vpx-interacting protein from differentiated human monocytic THP-1 cells that ectopically express tagged Vpx [21]. SAMHD1 is expressed in HIV-1 nonpermissive cell types, including THP-1 cells, primary monocytes, monocyte-derived macrophages and DCs, but not in HIV-1 permissive CD4^+^ T-cell lines [21]. Knockdown of endogenous SAMHD1 protein in non-permissive THP-1 cells and primary DCs alleviates HIV-1 restriction. By contrast, overexpression of SAMHD1 in monocytic U937 cells and then differentiation with PMA inhibits HIV-1 infection in the cells [21]. Independently, Hrecka and colleagues identified SAMHD1 from the human embryonic kidney cell line (HEK 293T) expressing tagged Vpx in a proteomic screen using multidimensional protein identification technology [22]. The authors further demonstrated that the inhibition of HIV-1 infection in monocyte-derived macrophages by Vpx-mediated proteasome degradation of SAMHD1 through the cellular E3 ubiquitin ligase complex [22]. Together, these studies confirmed that Vpx interacts with SAMHD1 and induces proteasomal degradation of SAMHD1 in THP-1 cells or macrophages, which can be restored by treatment with a proteasome inhibitor [21, 22]. A recent analysis further demonstrated that Vpx from HIV-2 or SIV engages SAMHD1 onto the E3 ubiquitin ligase complex CRL4/DCAF1 to initiate proteasomal degradation of SAMHD1 [49]. Moreover, Wei et al. identified a novel DCAF1-binding motif required for Vpx-mediated degradation of SAMHD1 protein [50], suggesting important interactions involved in the assembly of Vpx-hijacked DCAF1-DDB1-based E3 ubiquitin ligase complex. The molecular interactions between Vpx and SAMHD1 also suggest an evolutionary conflict between lentiviruses and host restriction factors, which lead to evolutionary studies of primate SAMHD1 and lentiviral Vpx proteins.

### 2.3. Evolutionary Biology of SAMHD1

SIVsm, SIVsm-derived SIV that infects rhesus macaques (SIVmac), and HIV-2 that infects humans encode both Vpr and Vpx proteins. By contrast, SIVcpz that naturally infects chimpanzees and HIV-1 that infects humans only encode Vpr, but not Vpx [44]. The *vpx* gene has been suggested to be derived from duplication of the primate lentivirus *vpr* gene, while these two related gene products may have different functions [44]. Several studies have demonstrated the importance of Vpx in SIV pathogenesis since macaques infected with Vpx-defective SIV had lower levels of viremia and viral replication, as well as slower AIDS progression compared to wild-type SIV-infected animals [51–53]. The important role of Vpx in lentiviral infection *in vivo* suggests that Vpx-mediated SAMHD1 degradation may facilitate SIV replication. However, it should be pointed out that Vpx may also have SAMHD1-independent effects in the infected cells, such as facilitating nuclear import of viral DNA [44]. Thus, the precise function of Vpx in lentiviral pathogenesis remains to be further investigated.

Evolutionary analysis of human and primate *SAMHD1* sequences revealed strong positive selection of the *SAMHD1 *genes [54–56], which is a common feature of all currently identified retroviral restriction factors [20]. Lim et al. reported that the ability of primate lentiviruses to degrade their host SAMHD1 proteins occurred prior to the birth of the lentiviral protein Vpx [55]. The authors observed that not only multiple Vpx but also some SIV Vpr proteins are able to degrade SAMHD1, which likely led to strong positive selection of SAMHD1 in the primate subfamily Cercopithecinae [55]. Thus, the authors suggested that the function of Vpr-mediated degradation of SAMHD1 occurred before the birth of the separated *vpx* gene in SIV, thereby initiating an evolutionary arms race between primate lentiviruses and SAMHD1.

**Figure 1:**
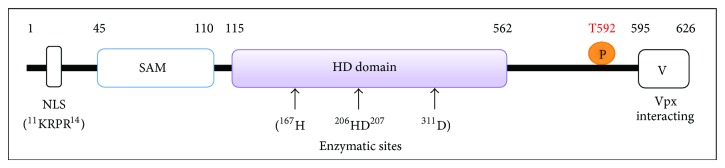
Schematic illustration of SAMHD1 protein and its functional domains. Numbers indicate amino acid positions of human SAMHD1 protein. The SAM domain, HD domain, and the C-terminal variable region of SAMHD1 are indicated. Other critical residues and motifs include nuclear localization signal (NLS), four critical residues in SAMHD1 NLS (^11^KRPR^14^), four residues in the enzymatic HD domain (H167, H206, D207, and D311), and phosphorylation of threonine at residue 529 (T592, indicated with the letter P).

Similarly, Laguette and colleagues performed evolutionary and functional analyses of the interactions between multiple primate SAMHD1 and lentiviral Vpx proteins and found that SAMHD1 restriction of HIV-1 is evolutionarily maintained among different primates, while antagonism of SAMHD1 by Vpx appears to be species-specific [54]. The authors also suggested that strong positive selection of SAMHD1 during primate evolution occurred in the Catarrhini ancestral branch before the separation between hominoids and Old World monkeys. Furthermore, the identification of SAMHD1 residues under positive selection guided mapping the short Vpx-interaction domain to the C-terminus of SAMHD1 [49, 54] (Figure 1). 

Using ancestral host state reconstruction and temporal dynamic analyses, Zhang and colleagues suggested that coevolution of primate SAMHD1 and lentivirus Vpx leads to the loss of the *vpx *gene in HIV-1 ancestor [56]. Their analyses indicated that the most recent common ancestor of SIV and HIV-1 was a SIV that had a *vpx* gene; however, the *vpx* gene of SIVcpz was lost approximately 3,643 to 2,969 years ago during the SIVcpz infection of chimpanzees [56]. HIV-1 therefore could not inherit the lost *vpx* gene from its ancestor SIV infecting chimpanzees. The lack of Vpx in HIV-1 likely results in restricted infection in myeloid cells that are important for antiviral immunity, which may contribute to the AIDS pandemic due to immune evasion of HIV-1 [56]. Taken together, these evolutionary and functional analyses of SAMHD1 and Vpx proteins not only confirmed the arms race between host restriction factors and lentiviruses, but also provided a pathogenic explanation why HIV-1 genome does not contain a *vpx *gene. It is conceivable that losing the *vpx *gene in HIV-1 ancestor might contribute to its cross-species transmission into humans and avoiding protective immune responses to viral infection.

### 2.4. Structure and Enzymatic Activities of Human SAMHD1 Protein

The HD domains in proteins have putative nucleotidase and phosphodiesterase activities [57], and the highly conserved histidine (H) and aspartic acid (D) residues in the HD domain of SAMHD1 are critical for its dNTPase activity [58]. Laguette et al. first showed that overexpression of the HD/AA (aa. 206-207) mutant SAMHD1 in U937 cells is not able to restrict HIV-1, suggesting that the phosphodiesterase activity of the HD domain is important for the restriction function of SAMHD1. Further analysis revealed that the HD domain of SAMHD1 is responsible for its dGTP-stimulated dNTPase activity [58, 59] (Figure 1).

The nuclear localization signal (NLS) of human SAMHD1 protein has been identified to residues ^11^KRPR^14^ in the N-terminus of the protein [60, 61]. Mutagenesis of these residues of SAMHD1 changed its nuclear distribution to the cytoplasm. SAMHD1 mutants localized to the cytoplasm can potently restrict HIV-1 and SIV similar to the wild type protein, but the nuclear Vpx proteins cannot degrade SAMHD1 mutants that localize to the cytoplasm [60, 61]. These studies suggest that cytoplasmic localization of SAMHD1 does not affect its dNTPase activity and anti-HIV function in nondividing cells. Of note, using confocal microscopy and subcellular fractionation techniques, Baldauf et al. demonstrated that endogenous SAMHD1 protein can localize in both the nucleus and cytoplasm in primary macrophages and CD4^+^ T-lymphocytes [24]. However, the physiological function of nuclear and cytoplasmic SAMHD1 remains unclear and it is unclear whether retroviral infection may alter the intracellular localization of SAMHD1 in primary monocytes, DCs, and macrophages.

### 2.5. *SMAHD1* Gene Expression and Regulation

*SAMHD1* mRNA is highly expressed in primary B-lymphocytes, CD4^+^ T-lymphocytes, and CD14^+^ monocytes isolated from healthy blood donors. Moreover, 2- to 6-fold variations of the levels of *SAMHD1 *mRNA were observed among different blood donors [62]. SAMHD protein expression has been reported in many types of primary immune cells from healthy donors, including B-cells, CD4^+^ and CD8^+^ T-cells, CD14^+^ monocytes, monocyte-derived macrophages, or DCs [21–25, 63–66]. However, the physiological function of SAMHD1 in these immune cell types remains unclear. Welbourn and colleagues identified naturally occurring splice variants of SAMHD1 in several cell lines, which lack exons 8-9 and 14, respectively [67]. These splice variants localize primarily to the nucleus and are sensitive to Vpx-dependent degradation. However, compared to full-length SAMHD1, these splice variants lack metabolic stability and catalytic activity to block HIV-1 infection [67]. Furthermore, the expression and potential role of the splice variants of SAMHD1 in HIV-1 primary target cells remain to be established.

A recent study by de Silva and colleagues reported that promoter methylation regulates SAMHD1 gene expression in human CD4^+^ T cells [65]. The *SAMHD1* promoter is methylated in CD4^+^ Jurkat and Sup-T1 T-cell lines, but not primary CD4^+^ T lymphocytes, which express high levels of SAMHD1. These data indicate a direct correlation between the methylation of the SAMHD1 promoter and transcriptional repression [65]. Further, SAMHD1 protein expression can be induced in CD4^+^ T cell lines by blocking the activity of DNA methyltransferase and histone deacetylase, suggesting that promoter methylation and histone deacetylation are epigenetic mechanisms by which regulate SAMHD1 gene expression [65]. Epigenetic mechanisms are critical for cancer initiation and progression through modulation of gene expression, and transcriptional gene repression via epigenetic modifications have been reported in many cancers [68]. Therefore, it is conceivable that downregulation of SAMHD1 to reduce its dNTPase activity can contribute to cancer development because cancer cells can maintain a high dNTP pool to support their rapid DNA replication and cell proliferation.

## 3. The Role of SAMHD1 in the Innate Immunity

The immunological function of SAMHD1 remains to be defined. *SAMHD1* mutations are involved in Aicardi-Goutières syndrome (AGS), a genetic encephalopathy mimicking congenital viral infection [69]. *SAMHD1* was first cloned from human DCs as an interferon (IFN)-*γ*-inducible gene [70] and has been proposed to act as a negative regulator of the IFN response [69]. Yan et al. reported that the cellular exonuclease TREX1 binds and degrades excess cytosolic HIV-1 DNA that could activate type I IFN expression and trigger innate immune responses against viral infection [71]. Thus, HIV-1 may evade innate immunity in the infected individuals through TREX1-mediated viral DNA degradation. It is possible that a similar role of SAMHD1 may play in HIV-1 immune evasion. Similar to *SAMHD1*, *TREX1* mutations in humans are associated with autoimmune and inflammatory diseases such as AGS [71]. It is currently unknown whether AGS patients are more susceptible to HIV-1 or other viral infections. Two studies demonstrated that peripheral blood mononuclear cells and CD4^+^ T-lymphocytes from AGS patients are more susceptible to HIV-1 infection *in vitro* compared to healthy donors' cells [23, 24]. Developing a *SAMHD1* knockout mouse model will likely provide a useful tool to study the role of SAMHD1 in the innate immunity and to examine whether SAMHD1 can block mouse retroviral infection or endogenous retrotransposons *in vivo*.

## 4. Potential Role of SAMHD1 in Blocking HIV-1 Infection *In Vivo*

Genetic analysis of the *SAMHD1 *gene polymorphisms has been applied to determine whether the expression of *SAMHD1* mRNA is affected by single nucleotide polymorphisms (SNPs) in *SAMHD1* and whether the SNPs are associated with HIV-1 infection status [62]. Using a tagging SNP approach, Coon et al. determined the association between eight tagging SNPs in the *SAMHD1* gene and the levels of *SAMHD1 *mRNA expression in 70 healthy white donors and identified one SNP that is significantly associated with *SAMHD1 *mRNA expression. However, analysis of the available dataset of the genome-wide association study including 857 HIV-1 controllers and 2,088 HIV-1 progressors from the European and African-American cohorts indicate that there is no significant association between SNPs in the *SAMHD1* gene and HIV-1 infection status [62]. It will be interesting and informative to analyze whether the polymorphism of *SAMHD1* is associated with HIV-2 infection or disease progression in humans.

In order to investigate the role of Vpx-mediated SAMHD1 antagonism in the virological and clinical outcome of HIV-2 infection, Yu et al. analyzed the SAMHD1 degradation activity of *vpx* alleles derived from seven viremic and four long-term aviremic HIV-2-infected individuals [72]. The authors found that the efficiency of Vpx-mediated SAMHD1 antagonism is not associated with the potency of viral control in HIV-2-infected individuals [72]. The functions of Vpx-mediated SAMHD1 degradation and enhancement of HIV-1 infection of myeloid cells are observed in most HIV-2-infected individuals including all seven patients who developed AIDS [72]. Although a small numbers of samples from HIV-2 infected individuals were analyzed, this study suggests that the activation of antiviral innate immune responses after Vpx-dependent infection of myeloid cells cannot fully explain less pathogenic effects in HIV-2-infected individuals who control viral replication and become long-term survivors.

SAMHD1-mediated HIV-1 restriction in resting CD4^+^ T-lymphocytes might be important for AIDS immunopathogenesis. Doitsh et al. reported that abortive HIV-1 reverse transcription in resting tonsil CD4^+^ T-lymphocytes causes proapoptotic cell death and proinflammatory response [73]. Of note, these detrimental immune responses after HIV-1 infection are triggered by accumulation of incomplete viral reverse transcripts, which likely contribute to the depletion of CD4^+^ T-cells in HIV-1 infected individuals [73]. It is currently unknown whether SAMHD1-mediated restriction of HIV-1 in resting CD4^+^ T-lymphocytes may trigger innate immune recognition of HIV-1 cDNA and thereby result in cell death and T-cell depletion [15].

HIV-1 cell-to-cell transmission is an efficient and rapid way to disseminate viral infection between DC and CD4^+^cells [10–12] or between CD4^+^ T-cells [74–76]. Puigdomenech et al. recently reported that SAMHD1 restricts HIV-1 cell-to-cell transmission and limits immune detection in monocyte-derived DCs [77]. This study was focused on HIV-1 transmission from infected CD4^+^ T-cells to SAMHD1-expressing or knocking-down DCs [77]. It is conceivable that SAMHD1-restriced HIV-1 infection in DCs would limit viral spreading from DCs to CD4^+^ T-cells, although the experimental evidence is lacking. More recently, Pauls et al. reported that upregulation of SAMHD1 in macrophages accounts for, at least in part, restriction of HIV-1 replication in macrophages by IL-12 and IL-18 [78]. This study suggests that cytokine-induced upregulation of SAMHD1 in macrophages or DCs can further protect these important antigen-presenting cells from direct HIV-1 infection *in vivo*. Additional *ex vivo* virological and immunological studies using myeloid cells and CD4^+^ T-cells from HIV-1 infected individuals are required to better understand the role of SAMHD1 in viral infection.

## 5. The Mechanisms by Which SAMHD1 Blocks Retroviral Infection in Cells

### 5.1. Limiting Retroviral Reverse Transcription by Decreasing Intracellular dNTP Pool

Previous studies using different retrovirus as a model system have demonstrated that intracellular nucleotide levels can regulate retroviral infection efficiencies in cells (reviewed in [79]). It has been known for over two decades that low levels of deoxynucleotides in peripheral blood lymphocytes could be a means to inhibit HIV-1 replication [80]. Efficient HIV-1 infection of macrophages depends on efficient cellular dNTP utilization by reverse transcriptase [81]. Likely due to low levels of the dNTP pool in resting cells, HIV-1 entry into quiescent primary lymphocytes can initiate reverse transcription of viral cDNA but cannot complete the full-length HIV-1 cDNA synthesis [82].

**Figure 2:**
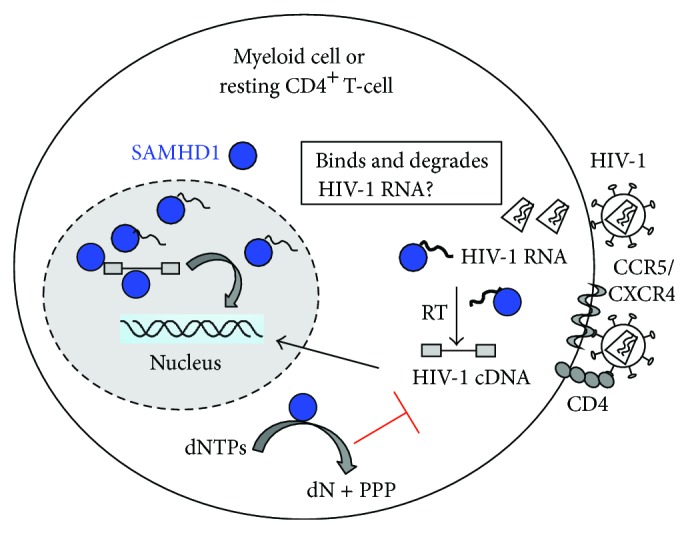
Proposed mechanisms of SAMHD1-mediated retroviral restriction. The diagram shows the proposed mechanisms by which SAMHD1 restricts HIV-1 infection in nondividing cells, such as myeloid cells and resting CD4^+^ T-cells. SAMHD1 possesses nuclease and dNTPase activities, which are dependent on its HD domain, but the SAM domain is required for maximal activity and nucleic acid binding. SAMHD1 functions as a dNTPase to decrease intracellular dNTP pool and thereby limits HIV-1 DNA synthesis during reverse transcription. Furthermore, SAMHD1 may directly bind and degrade retroviral RNA or viral DNA products generated in the infected cell.

Using purified recombinant SAMHD1 expressed in *E. coli*, Goldstone et al. and Powell et al. independently reported that SAMHD1 is a dGTP-stimulated dNTPase, which converts deoxynucleoside triphosphates (dNTPs) into to the constituent deoxynucleotides and inorganic triphosphate *in vitro* [58, 59]. Subsequent work by Lahouassa et al. indicated that SAMHD1 restricts HIV-1 replication in human macrophages by maintaining low levels of the intracellular dNTP pool [83]. SAMHD1-mediated HIV-1 restriction in resting CD4^+^ T-cells is also dependent on low dNTP levels in the cells [24]. Furthermore, St. Gelais and colleagues demonstrated that SAMHD1 restricts HIV-1 infection in monocyte-derived DCs by dNTP depletion since Vpx-mediated SAMHD1 degradation significantly increased intracellular dNTP concentrations and enhanced single-cycle and spreading HIV-1 infection in DCs [66]. These studies indicate a strong correlation between low levels of intracellular dNTPs and restriction of HIV-1 infection; however, addition of deoxynucleotides into resting CD4^+^ T-cells or macrophages to increase the intracellular dNTP pool cannot fully restore HIV-1 or SIV infections [24, 64], suggesting that decreasing the intracellular dNTP pool by SAMHD1 may not be the sole mechanism underlying retroviral restriction in nondividing cells (Figure 2). 

Gramberg and colleagues recently reported that prototype foamy virus (PFV) and human T cell leukemia virus type I (HTLV-1) are not restricted by human SAMHD1 [27]. Given that the reverse transcription of PFV mainly occurs before viral entry, which may not be affected by the dNTP level in the target cell. The complex human retrovirus HTLV-1 may have evolved a mechanism to counteract SAMHD1-mediated restriction, which may be due to a Vpx function-like viral protein encoded by HTLV-1 genome. Another possibility is that the reverse transcriptase of HTLV-1 may support the viral replication cycle in nondividing cells with low dNTP concentrations. Indeed, Jones et al. demonstrated that cell-free HTLV-1 can infect human blood myeloid DCs [84]. It would be interesting to compare HTLV-1 and HTLV-2 to examine whether HTLV-2 is sensitive to SAMHD1-mediated restriction function.

### 5.2. Phosphorylation of SAMHD1 Negatively Regulates Its HIV-1 Restriction Function

**Figure 3:**
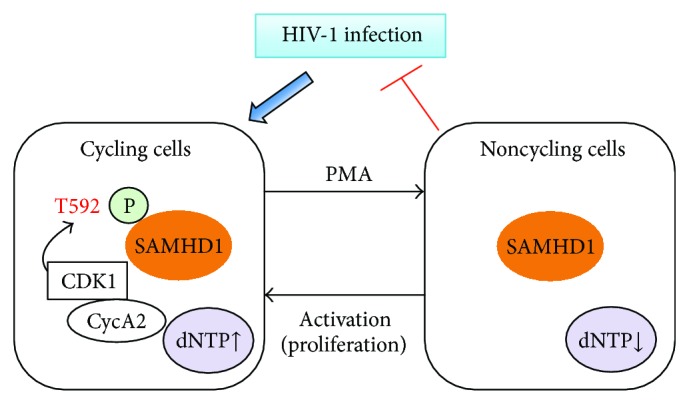
Phosphorylation of SAMHD1 negatively regulates its HIV-1 restriction function. SAMHD1 only exhibits antiretroviral activity when expressed in non-cycling cells because SAMHD1 is unphosphorylated in non-cycling cells. Phosphorylated threonine at residue 529 (T529) of human SAMHD1 is repressive for HIV-1 restriction in cycling cells. CDK1: cyclin-dependent kinase 1; CycA2: Cyclin A2. The letter P in the diagram indicates phosphorylation of T592 of human SAMHD1. PMA: phorbol 12-myristate 13-acetate.

Two recent studies reported that phosphorylation of SAMHD1 impairs its HIV-1 restriction function [85, 86]. White et al. showed that SAMHD1-mediated HIV-1 restriction function is regulated by the phosphorylation of threonine at residue 529 (T529); however, mutagenesis studies indicated that T529 phosphorylation of SAMHD1 does not affect its dNPTase activity [85]. Moreover, SAMHD1 contains a target motif for cyclin-dependent kinase 1 (CDK1) and CDK1 activity is responsible for SAMHD1 phosphorylation [85]. In agreement with these findings, Cribier et al. recently reported that phosphorylation of SAMHD1 by Cyclin A2/CDK1 regulates its HIV-1 restriction activity [86]. SAMHD1 phosphorylation regulates restriction, but not the cellular levels of dNTPs [85]. Thus, SAMHD1-mediated HIV-1 restriction may be independent of its dNTPase activity, suggesting additional cellular co-factors may contribute to SAMHD1-mediated HIV-1 restriction function (Figure 3). The role of Cyclin A2/CDK1 in regulating SAMHD1 anti-HIV-1 function remains to be investigated given that phosphorylation of T529 in human SAMHD1 does not affect its dNPTase activity [85]. One possibility is that phosphorylation of SAMHD1 may alter its structure or conformation and thereby regulate its HIV-1 restriction function.

### 5.3. Structure of SAMHD1 Can Affect Its HIV-1 Restriction Function

The crystal structure of the catalytic core of SAMHD1 reveals that the N-terminus truncated protein (aa. 110-626) is dimeric and indicates a structural basis for dGTP stimulation of catalytic activity hydrolyzing dNTPs [58]. The structure of full-length SAMHD1 has not solved, perhaps due to insolubility of the full-length protein. In contrast to the dimeric model suggested from the structural work [58], functional analyses of catalytic and anti-HIV activities of SAMHD1 have demonstrated that tetramerization of SAMHD1 is critical for its dNTPase activity and restriction of HIV-1 infection in nondividing cells [87]. By contrast, SAMHD1 mutagenesis studies by White et al. suggested that oligomerization of SAMHD1 is not correlated with its HIV-1 restriction activity [26, 85]. It is possible that distinct assays used in these studies may yield different results, which remains to be confirmed.

A recent study by Delucia et al. revealed that Vpx binding to SAMHD1 inhibits the catalytic activity of SAMHD1 before leading to SAMHD1 recruitment to the E3 ubiquitin ligase complex for proteasome-dependent degradation [88]. This study indicated that Vpx proteins interface with the C-terminus of primate SAMHD1 proteins with high affinity and this interaction inhibits SAMHD1 catalytic activity and induces disassembly of a dGTP-dependent oligomer [88]. Further studies of the structure and functionality of SAMHD1-Vpx complex will provide important insight into mechanisms underlying SAMHD1-mediated retroviral restriction.

### 5.4. Nuclease Activity of the Human SAMHD1 May Contribute to HIV Restriction

In addition to the dNTPase activity of SAMHD1, recent studies suggest that SAMHD1 can bind to and degrade HIV-1 mRNA, which can be a novel mechanism blocking retroviral infection [89–91]. Goncalves et al. identified SAMHD1 as a nucleic-acid-binding protein displaying a preference for RNA over DNA [89]. SAMHD1 is mislocalized due to AGS-associated mutations. Tungler et al. demonstrated that single-stranded RNA and DNA can promote the formation of the SAMHD1 complex [90]. They provided first direct evidence that SAMHD1 associates with endogenous nucleic acids *in situ*. Using fluorescence cross-correlation spectroscopy, the authors demonstrated that SAMHD1 specifically interacts with single-stranded RNA and DNA. They also found that nucleic-acid-binding and formation of SAMHD1 complexes appear to be mutually dependent. The interaction with nucleic acids and complex formation requires the HD domain and the C-terminal region of SAMHD1, but not the SAM domain. Furthermore, mutations associated with AGS exhibit both impaired nucleic acid-binding and complex formation implicating that interaction with nucleic acids is an important function of SAMHD1 [90]. These results highlight an important role of SAMHD1 in nucleic acid metabolism, which can associate with cell proliferation and cell cycle regulation.

Notably, Beloglazova et al. have reported exonuclease activity of the human SAMHD1 protein [91]. They found that purified full-length human SAMHD1 protein possesses metal-dependent 3′-5′exonuclease activity digesting single-stranded DNA and RNA substrates *in vitro*. In double-stranded DNA/RNA substrates, SAMHD1 preferentially cleaves 3′-overhangs and RNA in blunt-ended DNA/RNA duplexes. Full-length SAMHD1 also exhibits strong binding to DNA and RNA substrates with complex secondary structures, such as HIV-1* gag* and *tat* RNA or cDNA synthesized *in vitro*. The authors concluded that the nuclease and dNTPase activities of SAMHD1 are dependent on its HD domain, while the SAM domain is required for maximal exonuclease activity and nucleic acid binding [91].

Together, these biochemical analyses of the interactions between nucleic acids and SAMHD1 and its exonuclease activity suggest that SAMHD1 may directly bind to and degrade retroviral genomic RNA or transcribed viral mRNA, as well as viral cDNA products generated from reverse transcription in the infected cells. However, this hypothesis remains to be tested in HIV-1 infected cells. Further delineation of the mechanisms underlying SAMHD1-mediated HIV-1 RNA and DNA binding and degradation is important for fully understanding the HIV-1 restriction in non-cycling cells.

## 6. SAMHD1-Independent HIV-1 Restriction in Myeloid Cells and Resting CD4^+^ T-Cells

It is possible that additional cellular restriction factors expressed in myeloid cells can also contribute to blocking HIV-1 in these cells. Pertel et al. demonstrated that Vpx rescues HIV-1 transduction of DCs from the antiviral state caused by type 1 IFN independent of SAMHD1 [92]. In agreement with this finding, a recent study by Dragin et al. showed that IFN block to HIV-1 transduction in macrophages despite SAMHD1 degradation and high dNTP levels [93]. More recent studies also showed evidence for IFN alpha-induced but SAMHD1-independent cellular inhibitors of early HIV-1 infection in several cell lines [94]. Given that a diverse range of gene products are effectors of the type I IFN antiviral response [95], it is plausible that many other HIV restriction factors can account for the type I IFN-induced antiviral state in DCs. Thielen et al. suggest that deaminase activity of APOBEC3A isoforms in monocytes and macrophages may play an important role in restriction of HIV-1 infection in these cells [96]. Furthermore, APOBEC3A has been suggested to be a specific inhibitor of the early phases of HIV-1 infection in myeloid cells, likely functioning together with SAMHD1 to block HIV-1 replication with different mechanisms [97]. 

HIV-1 restriction in myeloid cells mainly occurs at the reverse transcription step due to SAMHD1; however, HIV-1 gene transcription in myeloid cells can be further blocked. Dong et al. have demonstrated blocking of HIV-1 gene expression at the transcriptional level due to lack of host factors that are required for viral gene transcription (such as cyclin T1) in primary undifferentiated monocytes or monocyte-derived DCs [98, 99]. These studies suggest that multifaceted mechanisms may contribute to HIV-1 restriction in human myeloid cells.

Additional host factor may contribute to HIV-1 restriction in resting CD4^+^ T-cells. For example, the host protein Murr1 involved in copper regulation can inhibit HIV-1 replication in unstimulated CD4^+^ T cells in part through its ability to inhibit basal and cytokine-stimulated nuclear factor-kappa B activity [100]. Goujon et al. also showed that HIV-1 infection can be blocked by type I IFN-induced proteins in primary macrophages and CD4^+^ T-cells [101]. Indeed, a diverse range of host proteins have been identified to act as effectors of the type I IFN antiviral response [95]. Moreover, a recent study revealed a novel anti-HIV mechanism within the innate immune response, in which Schlafen 11 (SLFN11) selectively inhibits viral protein synthesis in HIV-infected cells by means of codon-bias discrimination [102]. SLFN11 inhibits the late stages of HIV-1 production by preventing the expression of viral proteins. *Schlafen* genes are a subset of IFN-stimulated early response genes (ISGs) that are also induced directly by pathogens via the IFN regulatory factor 3 pathway [103]. It remains to be established whether SLFN11-mediated HIV-1 restriction plays a role in myeloid cells and resting CD4^+^ T-cells and whether HIV-1 has evolved a viral antagonist to counteract SLFN11.

## 7. Conclusion Remarks and Future Directions

The discovery of SAMHD1 as a host restriction factor blocking HIV-1 and other retroviruses has revealed novel molecular and cellular mechanisms to study intrinsic immunity against retroviruses and possibly other viral pathogens. New findings about mechanisms of SAMHD1-mediated HIV-1 restriction provide a platform towards better understanding the important role of SAMHD1 and its interactions with the viral antagonist (such as Vpx from SIVsm/HIV-2 and Vpr from some SIV lineages) in lentiviral pathogenesis. Further studies of SAMHD1 function and its mechanisms of blocking retroviral pathogens will benefit the design of more effective vaccines and antiretroviral interventions against HIV-1 infection. 

Manel and colleagues have suggested that HIV-1 restriction in DCs allows HIV-1 to avoid the antiviral immune responses derived from DCs, which are critical antigen presenting cells bridging the innate and adaptive immunity [48]. SAMHD1-mediated HIV-1 restriction in myeloid-lineage cells protects these cells from efficient HIV-1 infection, which likely prevents an innate immune response triggered by HIV-1 infection [29, 48]. By contrast, SIVsm and HIV-2 encode Vpx to overcome SAMHD1-mediated restriction, which likely induces protective innate immunity to confine viral infection in natural hosts. Thus, the interactions between SAMHD1 and Vpx may contribute to different consequences of HIV-1 and HIV-2 infection in humans. Future *in vivo* studies using animal models including macaque/SIV and humanized mouse/HIV-1 would be able to test this hypothesis.

How does an HIV-1 target cell sense viral infection and trigger the innate immune response is not fully understood and remains an important topic in the field [14, 29]. Studying retroviral restriction factors can help understand the mechanisms of anti-HIV innate immune responses and immune evasion. The retroviral restriction factor TRIM5 has been reported as an innate immune sensor for the retrovirus capsid lattice [104]. Galao et al. and Tokarev et al. have reported that the IFN-induced viral restriction factor tetherin stimulates nuclear factor-kappa B signaling and induces innate pro-inflammatory responses in cells [105, 106]. Recent studies by Wu and colleagues identified that cyclic GMP-AMP (guanosine monophosphate-adenosine monophosphate, or cGAMP) is a metazoan second messenger in innate immune signaling by cytosolic DNA [107]. Furthermore, it has been demonstrated that cellular cyclic GMP-AMP synthase (cGAS) is a cytosolic DNA sensor that activates the type I IFN pathway [108, 109]. It would be interesting to investigate whether cGAS can sense cytosolic HIV-1 DNA through the second messenger cGAMP in the infected cells and the potential role of SAMHD1 in this process. In summary, a better understanding of the mechanisms of host restriction factors and their additional physiological functions can facilitate the development of a more effective intervention against HIV-1 or other viral infections.

## Abbreviations

SAMHD1: Sterile alpha motif domain- and HD domain-containing protein 1
HIV-1: Human immunodeficiency virus type 1
HIV-2: Human immunodeficiency virus type 2
SIV: Simian immunodeficiency virus
DDB1: Damage-specific DNA binding protein 1
CUL4A: Cullin-4A
DCAF1: DDB1- and CUL4A-associated factor-1
DCs: Dendritic cells
APOBEC3G: Apolipoprotein B mRNA-editing, enzyme-catalytic, polypeptide-like 3G
TRIM5*α*: Tripartite motif-containing protein 5*α*.
